# Experimental study on the desorption effect of penetrant on gas-containing coal

**DOI:** 10.1371/journal.pone.0268684

**Published:** 2022-05-19

**Authors:** Dan Zhao, Baichen Liu, Zhongxin Liu, Chunguang Wang, Weiwei Su, Zhiyuan Shen

**Affiliations:** 1 College of Safety Science and Engineering, Liaoning Technical University, Fuxin, China; 2 Key Laboratory of Mine Power Disaster and Prevention of Ministry of Education, Huludao, Liaoning, China; 3 Coal Research Institute, Shenyang Research Institute, Ltd., Shenyang, Liaoning, China; 4 China State Key Laboratory of Coal Mine Safety Technology, Fushun, Liaoning, China; Tribhuvan University, NEPAL

## Abstract

Two non-ionic reagents, polyethylene glycol 4000 and Tween-80, two anionic reagents, sodium dodecyl benzenesulfonate and sodium lauryl sulfate, and a mixture of these non-ionic and anionic reagents were used as penetrants. The processes of replacement desorption and relief-pressure desorption of gas-containing coal were studied, the influence of the penetrant on the amount of gas replacement desorption and relief-pressure desorption was explored, and the change rule of the amounts of gas replacement desorption and relief-pressure desorption was analysed. The results show that the increase rate of the replacement desorption amount of the mixed penetrant is 11.81%-34.75%, and the decrease rate of the relief-pressure desorption amount is 51.68%-72.69%, which are higher values than those with a single penetrant. As the mass fraction of penetrant increases within the range of 0.5%~2%, the capacity of gas replacement desorption and hindering gas relief-pressure desorption will increase. At the same mass fraction, the effect of the mixed penetrant is better than that of the anionic penetrant, which in turn is better than that of the non-ionic penetrant.

## 1 Introduction

Gas outbursts and other problems in coal mines can easily lead to safety accidents, resulting in heavy casualties and economic losses [1–3]. The gas content can be effectively reduced by increasing the permeability of coal seam [4, 5]. Hydraulic measures such as water injection into coal seams [6], hydraulic fracturing [7, 8] and hydraulic punching [9] are often used to transform coal bodies in coal mine production sites to eliminate the risk of gas outbursts or improve the effect of gas extraction. It is of great significance to study the process of gas desorption to control gas release in coal mines and prevent outbursts. Scholars in China and abroad have conducted many studies on gas adsorption and desorption. Tao et al. [10] conducted isothermal adsorption experiments to reveal the influence of the first coalification jump on the adsorption capacity of coal. The results show that though the specific surface area of the candle coal is largely lower than that of the lignite, the CH4 adsorption capacity tends to decrease from the lignite to the candle coal due to material composition difference. Ye et al. [11] conducted methane isothermal adsorption/desorption experiments on coals with different rank, and found that the changes of heat and energy during methane adsorption and desorption are important factors affecting the production of coalbed methane. Zhang et al. [12] concluded that during gas desorption, the diffusion coefficient decreased linearly with increasing environmental pressure, and the magnitude of influence was related to time. Meng et al. [13] proposed that there was competitive adsorption between water and gas during desorption, and the interaction between coal molecules and water molecules was stronger. With the wide application of surfactants in the cleaning industry [14], sewage treatment [15] and other industries [16, 17], some scholars have tried to incorporate these compounds into the study of gas desorption. Peng et al. [18] studied the influence of soluble organic matter in coal on gas adsorption and desorption characteristics. The wetting effect of the penetrant solution at a certain concentration was better than that of pure water, and the effect was better in treating protruding coal seams. Abdulelah [19] found that the use of an anionic surfactant improved the wettability of the two shale samples during treatment, thus affecting the gas adsorption and desorption characteristics. Li et al. [20] concluded that the sample treated with the wettability reversal agent (12 fluoroalkyl trimethoxy silane) had the larger methane adsorption capacity than the wetted samples (sodium dodecyl benzene sulfonate, cocoanut fatty acid diethanolamide, and fatty alcohol-polyoxyethylene ether), with the latter facilitating the methane desorption. Li et al. [21] concluded that the application of surfactant can effectively enhance the water wettability of a coal matrix, and then promote methane desorption.

However, although the above research has achieved some promising results, the type of penetrant added to the coal seam is single and high pressure water injection can give a certain external force to the reagents, so that the experimental measurement results include two parts: the amount of gas desorption by penetrant and the amount of water injection pressure replacement gas, which is not conducive to observe the desorption effect of gas, so it is difficult to further explore the effect of penetrant on gas desorption. In this present study, the experimental method is improved to realize equal pressure water absorption of coal. Several different types of penetrants and their compound formulations are injected into coal seams to examine their effect on replacement and relief-pressure desorption through comparative analysis. Then the desorption rule of gas-containing coal was obtained. It is expected to provide reference for further understanding the influence mechanism of penetrants on gas desorption.

## 2 Experimental device and procedure

### 2.1 Experimental device design

**Fig 1:**
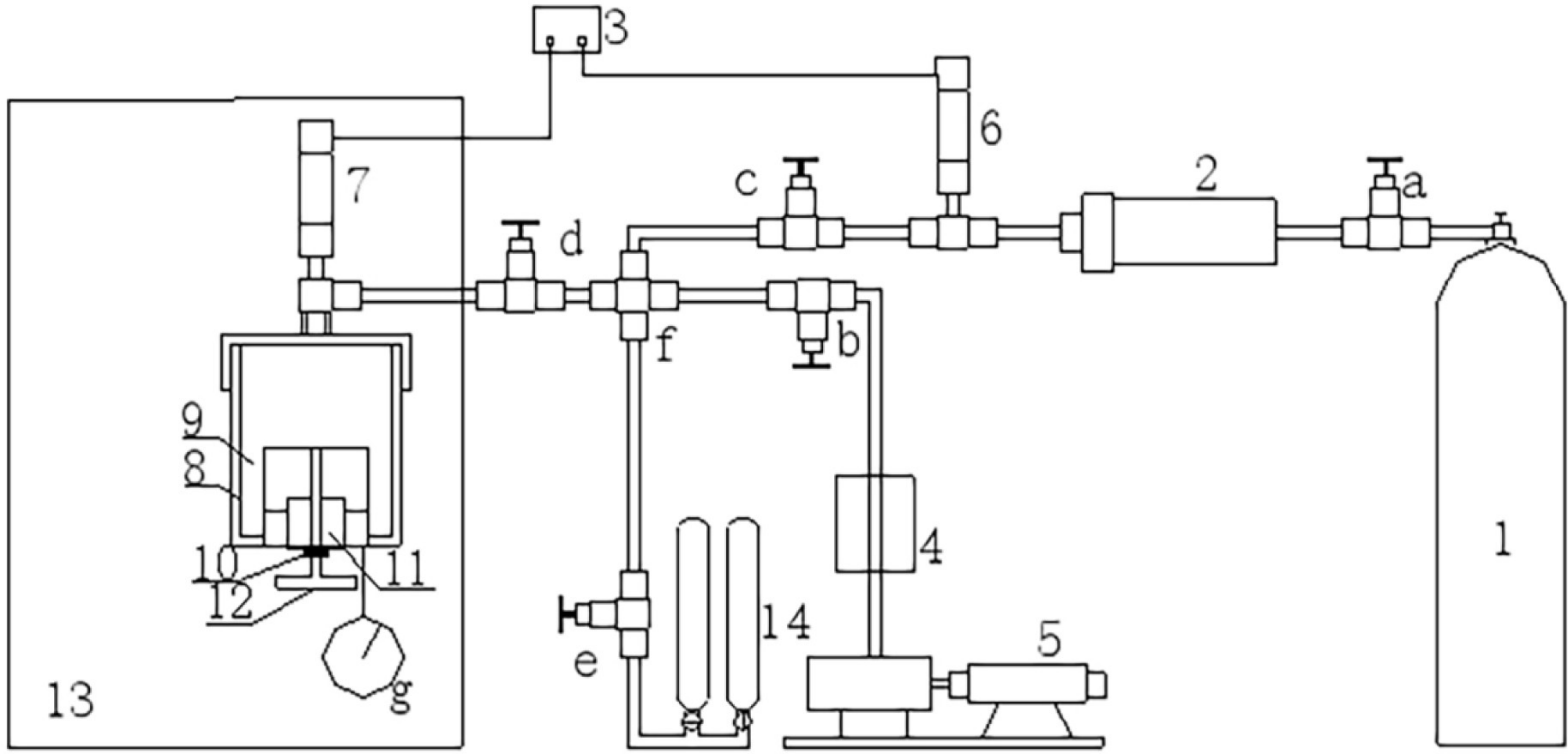
Schematic diagram of the experimental equipment. 1—High-pressure methane cylinders, 2—Reference tank, 3—Computer monitor, 4—Compound vacuum gauge, 5—Vacuum pump, 6~7—Pressure transducer, 8—Coal sample tank, 9—Coal, 10—Nut with internal rotation, 11—Built-in cylinders, 12—Needle valve, 13—Thermotank, 14—Desorption instrument, a~e—Valve, f—Cross, g—Pressure gauge.

The experimental equipment consisted of a vacuum degassing device, gas injection device, adsorption balance device, isobaric water injection device and desorption device. A schematic diagram of the experimental devices are shown in Fig 1. The coal samples used were all high metamorphic anthracite from Zhangminggou Coal Mine in northern Shaanxi. The results of the industrial analysis and parameter testing of coal samples are shown in Tables 1 and 2. Using these coal samples, the effect of using different penetrants applied under the same adsorption equilibrium pressure on gas replacement was studied.

**Table 1:** Industrial analysis and elemental analysis of the coal samples.

Coal Sample	Industry Analysis/w_t_%				Elemental Analysis/w_t_%				
	M	A	V	FC	C	H	N	O	S
Zhangminggou	3.70	15.10	29.01	52.02	79.10	4.50	1.35	14.65	0.24

M—Moisture, A—Ash content, V—Volatiles, FC—Fixed carbon.

**Table 2:** Ash analyses of the coal samples.

Coal Sample	Ash Content Analysis/w_t_%									
	SiO_2_	Al_2_O_3_	Fe_2_O_3_	CaO	MgO	TiO_2_	SiO_3_	K_2_O	Na_2_O	P_2_O_5_
Zhangmingggou	56.10	18.70	6.24	8.01	4.74	1.05	1.62	1.46	0.94	0.65

### 2.2 Experimental procedure

After testing the gas tightness of the device, the effect of penetrant type on gas desorption of the coal body was investigated. The experimental process was as follows:

1. Dry treatment of coal samples
  The coal samples were placed into a baking oven to dry at 105°C for 24 h. After drying, they were cooled in a closed drying device.
2. Addition of reagents to the water storage cylinder
  The cooled coal samples were taken out of the sealed device and weighed. Specific amounts of various regents were injected into the cylinder of the coal sample tank, the air of the water storage cylinder was discharged, and the screw was tightened to seal the injection opening.
3. Vacuum degassing treatment
  A water storage cylinder containing the coal samples was placed in an insulated water tank with a constant temperature water bath of 30°C. The cylinder was then connected them to a vacuum degassing device.
4. Adsorption balance treatment in air inflation
  An appropriate amount of methane gas with a concentration of 99% in the storage cylinder was injected into the reference tank, and the methane pressure in the reference tank and ambient temperature were recorded. The valve between the reference tank and the coal sample tank was opened, the methane gas was slightly regulated, the gas adsorption equilibrium pressure inside the coal sample tank was stabilized at 1 MPa, and the reference tank pressure and laboratory temperature were recorded before and after air inflation.
5. Isobaric adding water treatment
  After the adsorption balance, use the incompressibility of water, continue to tighten the inner screw nut to adjust the internal pressure of the built-in water storage cylinder, so that it is equal to the balance pressure of gas adsorption in the coal sample tank. The coal sample tank was inverted, and the water injection valve was opened in the coal sample tank to allow the reagent to naturally flow into the coal and complete water addition.
6. Replacement desorption test
  The automatic monitoring system monitored the gas pressure of the coal sample tank in real time, and automatically recorded changes in the gas pressure. When the pressure value in the coal sample tank was stable, the gas in the coal sample tank was balanced again.
7. Relief-pressure desorption test
  The valve between the coal sample tank and the desorption measuring device was opened. A gas bag was used to collect the gas discharged from the pressure relief of the coal sample tank. When the replacement pressure of gas in the coal sample tank dropped to standard atmospheric pressure, the tee was rotated and the valve was closed. At the same time, a stopwatch was started to record the time required to replace the gas inflow flowmemter in the desorption device. The desorption experiment was conducted at standard atmospheric pressure for 2 h.

## 3 Experimental results

### 3.1 Determination of the amount of gas replacement desorption

According to the gas pressure data recorded in the experiment, the amount of gas replacement desorption was quantified, and the effects of gas replacement with different penetrants were analysed. The specific calculation method was as follows:

The gas pressure in the reference tank and ambient temperature were recorded. Then, the equilibrium equation of methane in the reference tank before and after air inflation was:

$$\begin{array}{l} P_{cq}V_{0}=Z_{cq}n_{cq}RT_{cq}\\ P_{ch}V_{0}=Z_{ch}n_{ch}RT_{ch} \end{array}$$ (1)

In the formula, *Pcq* is the reference tank pressure before air inflation, Pa. *Pch* is the reference tank pressure after air inflation, Pa. *V*_*0*_ is the free volume of the reference tank and pipeline, mL. *n*_*cq*_ is the number of moles of methane in the reference tank before air inflation, mol. *n*_*ch*_ is the number of moles of methane in the reference tank after air inflation, mol. *T*_*cq*_ is the temperature in the laboratory before air inflation, K. *T*_*ch*_ is the temperature in the laboratory after air inflation, K. *Z*_*cq*_ is the compression factor of methane gas under *T*_*cq*_ before air inflation and is dimensionless. *Z*_*ch*_ is the compression factor of methane gas under *T*_*ch*_ after air inflation and is dimensionless. *R* is the molar gas constant, J.mol^-1^.K^-1^.

The reduction in the amount of gas in the reference tank is calculated as the total amount of gas filled in the coal sample tank. Eq (1) can be used to obtain:

$$\Delta n=n_{cq}-n_{ch}=\frac{V_{0}}{R}(\frac{P_{cq}}{Z_{cq}T_{cq}}-\frac{P_{ch}}{Z_{ch}T_{ch}})$$ (2)

Then the total volume *Q*_*c*_ of the methane filled is:

$$Q_{c}=\Delta V=\Delta n\times 22.4\times 1000=\frac{22400}{R}(\frac{P_{cq}}{Z_{cq}T_{cq}}-\frac{P_{ch}}{Z_{ch}T_{ch}})$$ (3)

In this equation, *Q*_*c*_ is the total volume of methane, mL/g, and 22.4 refers to the volume of 1 mole of methane under standard temperature and pressure, L.

It is assumed that the volume of the remaining free space in the coal sample tank remains unchanged after the water in the cylinder wets the coal body. Then the gas replaced by the penetrant injected into the coal sample tank is rebalanced, and the free volume *V*_*sh*_ in the coal sample tank is:

$$V_{sh}=V_{f}$$ (4)

$$\begin{array}{c} P_{sq}V_{f}=Z_{sq}n_{sq}RT_{0}\\ P_{sh}V_{sh}=Z_{sh}n_{sh}RT_{0} \end{array}$$ (5)

In the equation, *P*_*sq*_ is the adsorption equilibrium pressure in the coal sample tank before adding water, MPa. *P*_*sh*_ is the equilibrium pressure in the coal sample tank after the replacement is over, MPa. *V*_*f*_ is the remaining free volume of the coal sample tank before adding water, cm^3^. *V*_*sh*_ is the remaining free volume of the coal sample tank after the replacement is over, cm^3^. *n*_*sq*_ is the number of moles of free methane in the coal sample tank before adding water, mol. *n*_*sh*_ is the number of moles of free methane in the coal sample tank after the replacement is complete. *T*_*0*_ is the temperature in the constant temperature water bath where the coal sample tank is located, K. *Z*_*sq*_ is the compression factor of methane before adding water and is dimensionless. *Z*_*sh*_ is the compression factor of methane after the replacement is over and is dimensionless.

Before the penetrant is injected, the volume *Q*_*sq*_ of free gas in the free space of the coal sample tank in the equilibrium state under standard temperature and pressure is:

$$Q_{sq}=22400n_{sq}=\frac{22400V_{f}P_{sq}}{mRT_{0}Z_{sq}}$$ (6)

In the equation, *Q*_*sq*_ is the free gas volume under standard temperature and pressure, mL/g.

Then before the penetrant is injected, the gas volume *Q*_*xf*_ in the adsorbed state is:

$$Q_{xf}=Q_{c}-Q_{sq}$$ (7)

The coal was wet to replace the methane in the adsorbed state in the coal sample tank, and increase in the amount of the replaced gas is the mashgas replacement desorption amount, which can be obtained from Eq (5):

$$\Delta n=n_{zh}-n_{sh}=\frac{1}{RT_{0}}(\frac{P_{sh}V_{sh}}{Z_{zh}}-\frac{P_{sq}V_{f}}{Z_{sq}})$$ (8)

Combined with Eq (4), it can be concluded that:

$$\Delta n=\frac{V_{f}}{RT_{0}}(\frac{P_{sq}}{Z_{sq}}-\frac{P_{sq}}{Z_{sq}})$$ (9)

Then the gas replacement desorption amount *Q*_*zh*_ is:

$$Q_{zh}=\frac{22400V_{f}}{mRT_{0}}(\frac{P_{sh}}{Z_{sh}}-\frac{P_{sq}}{Z_{sq}})$$ (10)

Combined with Formulas (1) ~ (10), the replacement desorption amount of the gas-containing coal body injected with penetrant was calculated and further comparative analysis was performed.

### 3.2 Results of replacement desorption experiment

**Fig 2:**
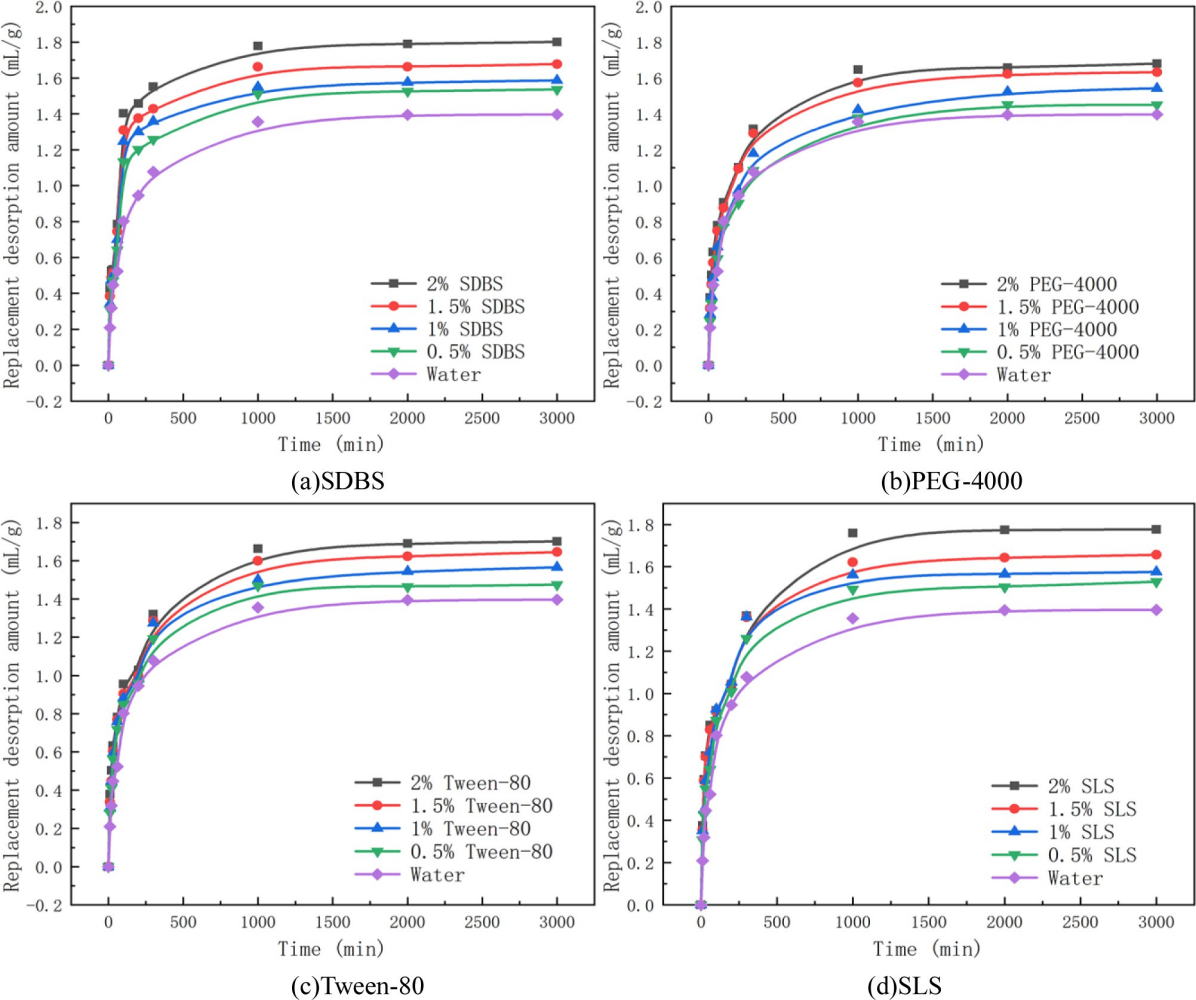
Changes in the replacement desorption amount caused by a single penetrant. (a) SDBS. (b) PEG-4000. (c) Tween-80. (d) SLS.

**Fig 3:**
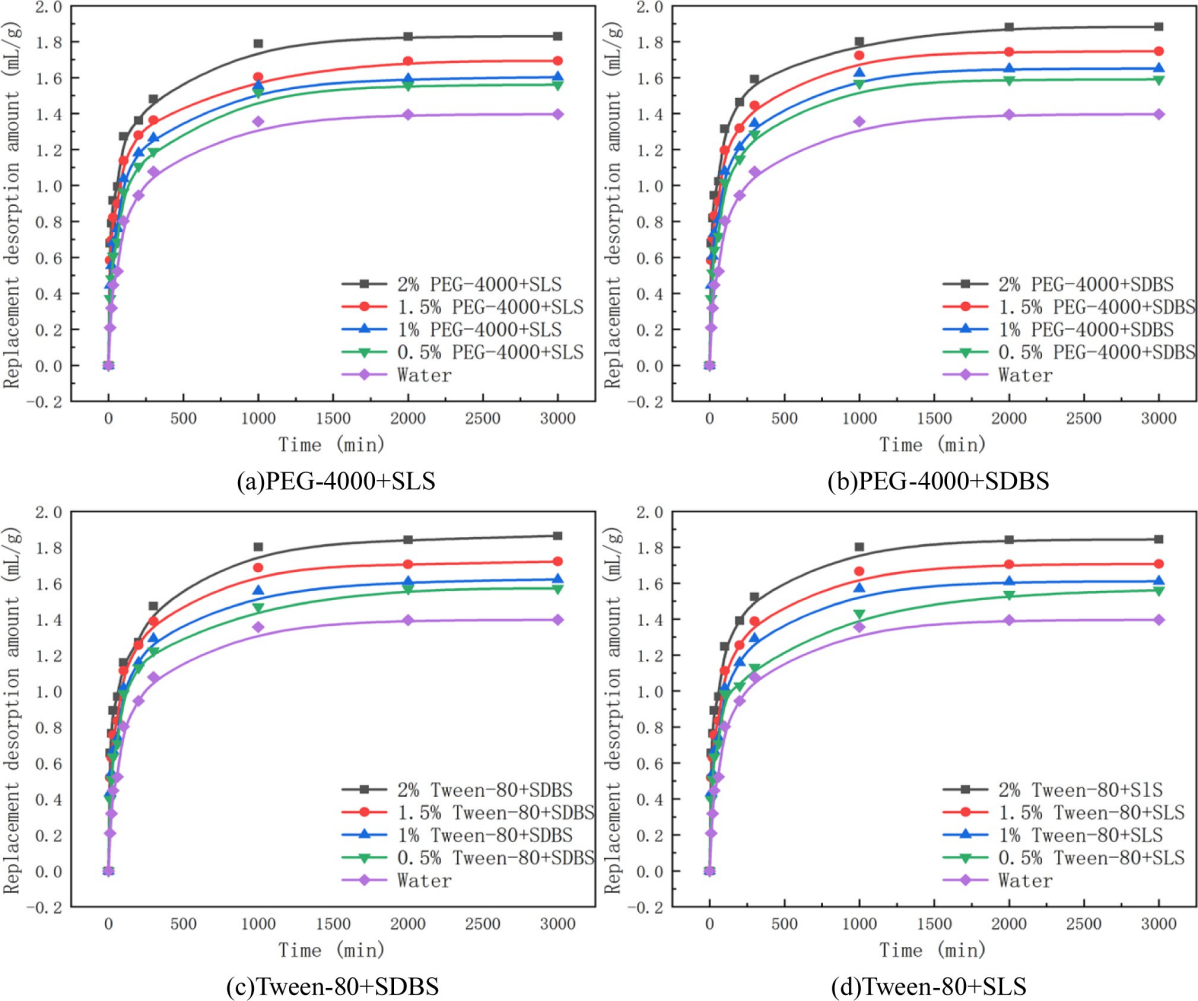
Changes in the replacement desorption amount caused by mixed penetrants. (a) PEG-4000+SLS. (b) PEG-4000+SDBS. (c) Tween-80+SDBS. (d) Tween-80+SLS.

To more accurately describe the influence of penetrants on the replacement of gas-containing coal body, using Eq (10), the amount of gas replacement desorption by penetrants with different mass fractions under the adsorption equilibrium pressure of 1 MPa was calculated. The results are shown in Table 3. The increasing range is the comparison of the amount of gas replacement desorption by penetrants and that of gas replacement desorption by water and is presented in Table 3. The change curves of the replacement desorption amount with time are shown in Figs 2 and 3.

**Table 3:** The replacement desorption amount of different penetrants with different mass fractions.

Penetrant	Mass fraction(%)	Weight of the coal sample(g)	Amount of adsorbed gas(mL/g)	Amount of replaced gas(mL/g)	Increasing range(%)
SDBS	2	219.86	7.81	1.80	29.01
	1.5	220.62	7.80	1.68	20.16
	1	220.85	7.77	1.59	13.64
	0.5	221.94	7.75	1.54	10.04
PEG-4000	2	220.99	7.78	1.68	20.33
	1.5	220.08	7.79	1.63	16.97
	1	221.42	7.77	1.54	10.48
	0.5	220.22	7.79	1.45	3.88
Tween-80	2	220.99	7.76	1.70	21.85
	1.5	220.62	7.75	1.65	17.84
	1	220.07	7.75	1.57	12.14
	0.5	220.57	7.77	1.48	2.65
SLS	2	219.92	7.79	1.78	27.26
	1.5	220.22	7.78	1.66	18.66
	1	220.23	7.78	1.57	12.84
	0.5	220.53	7.77	1.53	9.54
PEG-4000+SLS	2	220.17	7.80	1.82	30.44
	1.5	220.86	7.76	1.69	21.12
	1	221.03	7.72	1.60	14.29
	0.5	221.32	7.73	1.56	11.81
PEG-4000+SDBS	2	220.31	7.71	1.88	34.75
	1.5	220.64	7.78	1.74	24.71
	1	220.25	7.75	1.65	17.86
	0.5	220.33	7.81	1.59	13.96
Tween-80+SDBS	2	221.32	7.80	1.86	33.31
	1.5	219.93	7.72	1.72	23.28
	1	220.79	7.78	1.62	15.71
	0.5	220.95	7.77	1.57	12.53
Tween-80+SLS	2	220.69	7.71	1.84	31.88
	1.5	220.18	7.76	1.70	21.85
	1	220.35	7.77	1.61	15.02
	0.5	220.03	7.75	1.56	11.82

### 3.3 Results of relief-pressure desorption experiment

**Fig 4:**
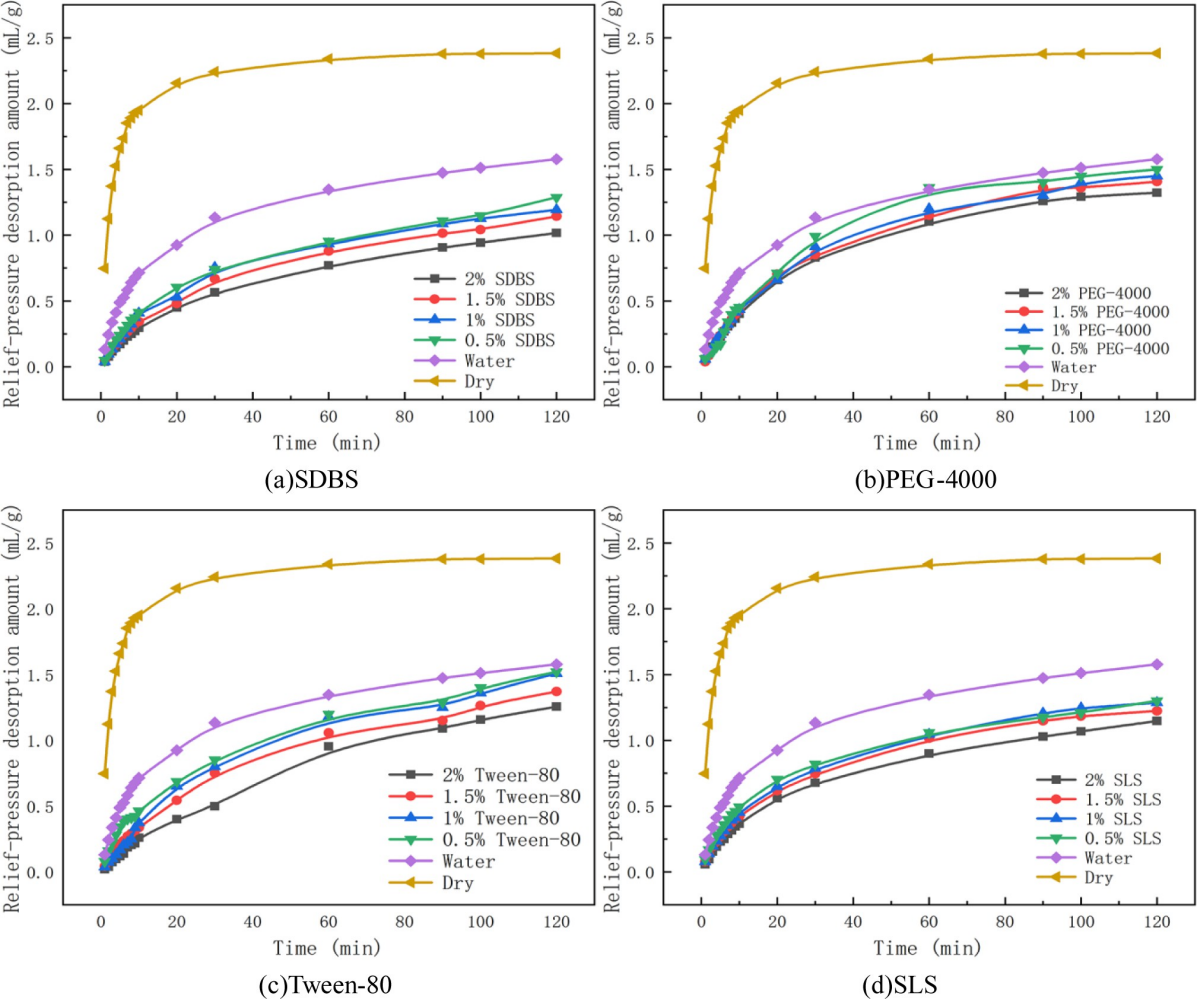
Variation in gas relief-pressure desorption by a single penetrant. (a) SDBS. (b) PEG-4000. (c) Tween-80. (d) SLS.

**Fig 5:**
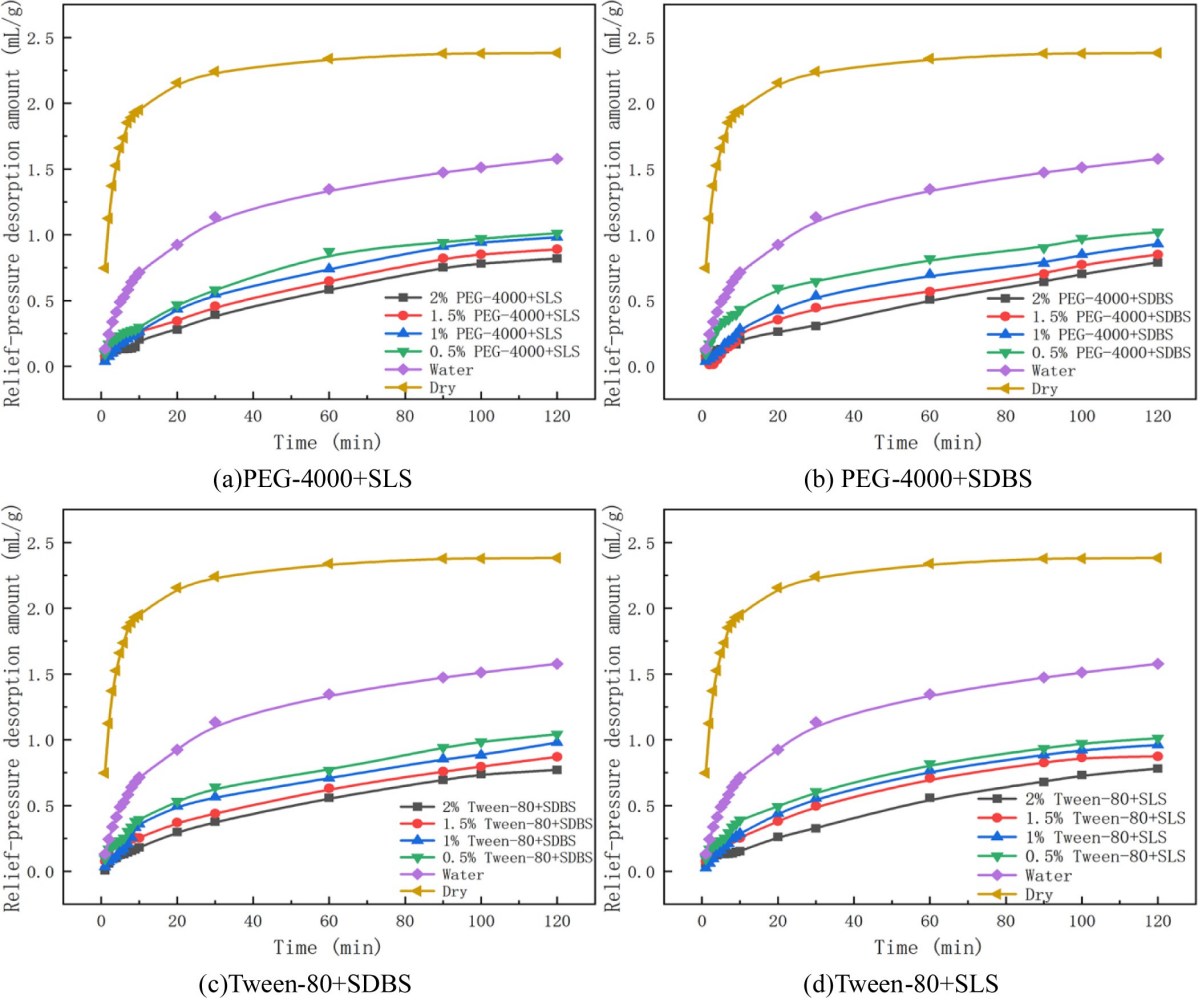
Variation in gas relief-pressure desorption by mixed penetrants. (a) PEG-4000+SLS. (b) PEG-4000+SDBS. (c) Tween-80+SDBS. (d) Tween-80+SLS.

The process of gas relief-pressure desorption in coal was studied under an adsorption equilibrium pressure of 1 MPa. The changes in gas desorption amount with time were measured in the experiment at 2 h after the pressure was relieved, as shown in Figs 4 and 5 and Table 4. The amount of gas relief-pressure desorption by water into coal samples was 1.58 mL/g, and that of dry coal samples was 2.38 mL/g. The decreasing range (water) is the comparison of the amount of gas relief-pressure desorption amount by penetrants and that obtained by using water, and the decreasing range (dry) is the comparison of the amount of gas relief-pressure desorption obtained by penetrants and that obtained under dry conditions, as presented in Table 4.

**Table 4:** Relief-pressure desorption amount of different penetrants with different mass fractions.

Penetrant	Mass fraction (%)	The amount of gas relief-pressure desorption (mL/g)	Decreasing range(water) (%)	Decreasing range(dry) (%)
SDBS	2	1.02	35.55	57.32
	1.5	1.14	27.55	52.02
	1	1.19	24.38	49.92
	0.5	1.29	18.39	45.96
PEG-4000	2	1.32	16.12	44.45
	1.5	1.41	10.85	40.96
	1	1.45	8.10	39.13
	0.5	1.50	4.90	37.03
Tween-80	2	1.25	20.39	47.27
	1.5	1.37	13.11	42.46
	1	1.51	4.60	36.81
	0.5	1.52	3.50	36.12
SLS	2	1.15	27.30	51.85
	1.5	1.22	22.43	48.63
	1	1.29	18.44	45.99
	0.5	1.30	17.57	45.41
PEG-4000+SLS	2	0.82	48.10	65.55
	1.5	0.89	43.67	62.61
	1	0.98	37.80	58.81
	0.5	1.01	36.07	57.56
PEG-4000+SDBS	2	0.79	50.01	66.81
	1.5	0.85	46.20	64.29
	1	0.93	41.06	60.97
	0.5	1.02	35.44	57.14
Tween-80+SDBS	2	0.77	51.27	67.65
	1.5	0.87	44.94	63.45
	1	0.98	37.90	58.88
	0.5	1.04	34.18	56.30
Tween-80+SLS	2	0.78	50.63	67.22
	1.5	0.87	44.94	63.45
	1	0.96	39.05	59.63
	0.5	1.01	36.08	57.56

## 4 Experimental discussion

### 4.1 Discussion of replacement desorption experiment

Based on the replacement desorption amount of penetrants with different mass fractions presented in Table 3, Figs 2 and 3, the influence of penetrants on the replacement desorption amount of gas-containing coal body was analyzed. After the penetrants wetted the gas-containing coal, rapid replacement and slow replacement took place. The self-priming effect of the coal forced water molecules to enter the coal, and the water molecules entered the holes through the crannies, resulting in a rapid increase in the replacement desorption amount of gas. Over time, the effect of the capillary force gradually decreased, and the degree of water entering the holes of coal body openings weakened. Finally, wetting ceased, and the gas in the coal sample tank reached gaseous equilibrium.

When the coal samples are in the same condition of adsorption equilibrium, there is little difference in the adsorption amount of gas among dry coal samples. The replacement desorption amount of gas is related to the mass fraction of the penetrant, and the larger the mass fraction is, the greater the replacement amount of gas will be at the same time. By analysing the process of replacement, it was found that the gas replacement by penetrant occured mainly within the first 1000 min of coal water absorption. At this stage, the gas replacement desorption amount accounts for 91.67%~98.73% of the total replacement desorption amount.The amount of gas replaced by penetrant is 1.45~1.88 mL/g. The replacement of gas by water occured mainly within the first 1000 min. The amount of gas replacement desorption by water accounted for approximately 96.58% of the total amount.The total amount of water replacement gas is approximately 1.39 mL/g. Based on the amount of gas replacement, the wetting effect of penetrant is better than that of water, and is most obvious in the initial stage.

As seen from the data in Table 3, with the increasing mass fraction of penetrant, the amount of gas replacement desorption and the capacity of gas replacement of penetrant are also increased. Compared with that achieved with water, the amount of gas replacement desorption increased by 3.88%~34.75% after the penetrant contacted the coal.Among the penetrants used, the increasing ranges of the amount of gas replacement desorption by anionic solutions were 9.54%-29.01%. The anionic groups in the anionic penetrant repel with the anionic groups in the coal, and the hydrophobic groups tend to adsorb on the surface of the coal, while the anionic groups face outwards, which increases the repulsion between the coal particles and the pore wall and promotes the dispersion of the particles to migrate and replace the gas in the coal. The increasing ranges of the amount of gas replacement desorption by non-ionic solutions were 3.88%-21.85%. The non-ionic penetrant solution does not contain groups that repel the anionic groups on the surface of coal, so its gas replacement ability is slightly worse than that of the anionic solution.The increase rate of the amount of gas replacement desorption by mixed penetrant solution was 11.81%-34.75%. Because of the non-ideal surface phase and micelle phase of the mixed penetrant solution, a synergistic effect of the mixed solution occurs. Therefore, under equivalent mass fraction conditions, the increase rate of the mixed penetrant solution is higher than that of the anionic solution, so the gas replacement capacity of the mixed penetrant solution is better than that of the anionic solution.

### 4.2 Discussion of relief-pressure desorption experiment

As shown in Figs 4 and 5 and Table 4, compared with that in the absence of an external liquid, the amount of gas relief-pressure desorption decreased after the solutions intruded into the coal. This finding indicates that during relief-pressure desorption, water enters the coal body and forms capillary resistance in its pores. As the pressure difference between the inside and outside of the pores is not enough to overcome the resistance, it is difficult for the gas to move outward, resulting in a water-locking effect. It can be seen from the curves of gas relief-pressure desorption that this process consists of two stages: rapid desorption and slow desorption. After a long time, the desorption stopped.

With increasing mass fraction of penetrant, the amount of relief-pressure desorption decreased gradually, and the capacity of hindering gas relief-pressure desorption increased gradually. By analysing the experimental data of the amount of gas relief-pressure desorption, it can be seen that compared with the amount of gas relief-pressure desorption obtained using water, that obtained using penetrants is 3.5%~51.27% less. This value is 36.12%~67.65% less than the amount of gas relief-pressure desorption under dry conditions.Among the penetrants applied under dry conditions, the amount of gas relief-pressure desorption in anionic solutions was 45.41%~57.32% less. Anionic solutions have the characteristics of reducing the water surface tension and contact angle, but increasing the capillary resistance and hindering gas desorption.The amount of gas relief-pressure desorption with non-ionic solution was 36.12%~47.27% less than that with the anionic solution. Compared with anionic solutions, non-ionic solutions perform slightly worse at reducing the surface tension and contact angle, so their ability to hinder gas desorption is slightly worse than that of anionic solutions. The amount of gas relief-pressure desorption obtained using the mixed penetrants was 56.30%~67.65% less than that of single penetrants. Because of its synergistic effect, the mixed solution mediates a stronger desorption effect than the anionic solution. Compared with the anionic solution of the same mass fraction, the mixed solution has a stronger ability to reduce gas relief-pressure desorption.

## 5 Conclusion

1. The penetrant have a replacement effect on the gas in the coal. As the mass fraction of penetrant increased from 0.5%~2%, the gas replacement desorption capacity of the penetrant increased. The amounts of gas replaced by the penetrant were 1.45~1.88 mL/g.
2. In the process of relief-pressure desorption, the amount of gas desorption of coal containing penetrant is less than that of dry coal sample at the same time. As the mass fraction of penetrant increased from 0.5%~2%, the ability of hindering gas relief-pressure desorption increased. The amount of gas relief-pressure desorption by the penetrant was 0.65~1.52 mL /g.
3. At the same mass fraction, the capacity of gas replacement desorption and hindering gas relief-pressure desorption of the mixed penetrant is the best, anionic solutions perform slightly worse, and the non-ionic penetrant solution was the worst.

Since the experimental test system is closed, the gas pressure in the coal sample tank will increase the adsorption capacity of gas during the replacement, thus affecting the replacement effect. In the future, it is necessary to study the replacement strength of gas by penetrant solution under constant pressure.

## Supporting information

S1 File (DOCX)
