# Supplementary material for: Experimental study on the desorption effect of penetrant on gas-containing coal
Source: PLoS One. 2022 May 19;17(5):e0268684. doi: 10.1371/journal.pone.0268684 (PMC9119538; doi:10.1371/journal.pone.0268684)
Supplement: S1 File — (DOCX) [file pone.0268684.s001.docx]

**Table 1. Industrial analysis and elemental analysis of the coal samples**

| Coal Sample | Industry Analysis/w_t_% | | | | Elemental Analysis/w_t_% | | | | |
| --- | --- | --- | --- | --- | --- | --- | --- | --- | --- |
|  | M | A | V | FC | C | H | N | O | S |
| Zhangminggou | 3.70 | 15.10 | 29.01 | 52.02 | 79.10 | 4.50 | 1.35 | 14.65 | 0.24 |

**Table 2. Ash analyses of the coal samples**

| Coal Sample | Ash Content Analysis/w_t_% | | | | | | | | | |
| --- | --- | --- | --- | --- | --- | --- | --- | --- | --- | --- |
|  | SiO_2_ | Al_2_O_3_ | Fe_2_O_3_ | CaO | MgO | TiO_2_ | SiO_3_ | K_2_O | Na_2_O | P_2_O_5_ |
| Zhangmingggou | 56.10 | 18.70 | 6.24 | 8.01 | 4.74 | 1.05 | 1.62 | 1.46 | 0.94 | 0.65 |

**Table 3. The replacement desorption amount of different penetrants with different mass fractions**

| Penetrant | Mass fraction(%) | Weight of the coal sample(g) | Amount of adsorbed gas(mL/g) | Amount of replaced gas(mL/g) | Increasing range(%) |
| --- | --- | --- | --- | --- | --- |
| SDBS | 2 | 219.86 | 7.81 | 1.80 | 29.01 |
|  | 1.5 | 220.62 | 7.80 | 1.68 | 20.16 |
|  | 1 | 220.85 | 7.77 | 1.59 | 13.64 |
|  | 0.5 | 221.94 | 7.75 | 1.54 | 10.04 |
| PEG-4000 | 2 | 220.99 | 7.78 | 1.68 | 20.33 |
|  | 1.5 | 220.08 | 7.79 | 1.63 | 16.97 |
|  | 1 | 221.42 | 7.77 | 1.54 | 10.48 |
|  | 0.5 | 220.22 | 7.79 | 1.45 | 3.88 |
| Tween-80 | 2 | 220.99 | 7.76 | 1.70 | 21.85 |
|  | 1.5 | 220.62 | 7.75 | 1.65 | 17.84 |
|  | 1 | 220.07 | 7.75 | 1.57 | 12.14 |
|  | 0.5 | 220.57 | 7.77 | 1.48 | 2.65 |
| SLS | 2 | 219.92 | 7.79 | 1.78 | 27.26 |
|  | 1.5 | 220.22 | 7.78 | 1.66 | 18.66 |
|  | 1 | 220.23 | 7.78 | 1.57 | 12.84 |
|  | 0.5 | 220.53 | 7.77 | 1.53 | 9.54 |
| PEG-4000+SLS | 2 | 220.17 | 7.80 | 1.82 | 30.44 |
|  | 1.5 | 220.86 | 7.76 | 1.69 | 21.12 |
|  | 1 | 221.03 | 7.72 | 1.60 | 14.29 |
|  | 0.5 | 221.32 | 7.73 | 1.56 | 11.81 |
| PEG-4000+SDBS | 2 | 220.31 | 7.71 | 1.88 | 34.75 |
|  | 1.5 | 220.64 | 7.78 | 1.74 | 24.71 |
|  | 1 | 220.25 | 7.75 | 1.65 | 17.86 |
|  | 0.5 | 220.33 | 7.81 | 1.59 | 13.96 |
| Tween-80+SDBS | 2 | 221.32 | 7.80 | 1.86 | 33.31 |
|  | 1.5 | 219..93 | 7.72 | 1.72 | 23.28 |
|  | 1 | 220.79 | 7.78 | 1.62 | 15.71 |
|  | 0.5 | 220.95 | 7.77 | 1.57 | 12.53 |
| Tween-80+SLS | 2 | 220.69 | 7.71 | 1.84 | 31.88 |
|  | 1.5 | 220.18 | 7.76 | 1.70 | 21.85 |
|  | 1 | 220.35 | 7.77 | 1.61 | 15.02 |
|  | 0.5 | 220.03 | 7.75 | 1.56 | 11.82 |

**Table 4. Relief-pressure desorption amount of different penetrants with different mass fractions**

| Penetrant | Mass fraction（%） | The amount of gas relief-pressure desorption（mL/g） | Decreasing range(water)（%） | Decreasing range(dry)（%） |
| --- | --- | --- | --- | --- |
| SDBS | 2 | 1.02 | 35.55 | 57.32 |
|  | 1.5 | 1.14 | 27.55 | 52.02 |
|  | 1 | 1.19 | 24.38 | 49.92 |
|  | 0.5 | 1.29 | 18.39 | 45.96 |
| PEG-4000 | 2 | 1.32 | 16.12 | 44.45 |
|  | 1.5 | 1.41 | 10.85 | 40.96 |
|  | 1 | 1.45 | 8.10 | 39.13 |
|  | 0.5 | 1.50 | 4.90 | 37.03 |
| Tween-80 | 2 | 1.25 | 20.39 | 47.27 |
|  | 1.5 | 1.37 | 13.11 | 42.46 |
|  | 1 | 1.51 | 4.60 | 36.81 |
|  | 0.5 | 1.52 | 3.50 | 36.12 |
| SLS | 2 | 1.15 | 27.30 | 51.85 |
|  | 1.5 | 1.22 | 22.43 | 48.63 |
|  | 1 | 1.29 | 18.44 | 45.99 |
|  | 0.5 | 1.30 | 17.57 | 45.41 |
| PEG-4000+SLS | 2 | 0.82 | 48.10 | 65.55 |
|  | 1.5 | 0.89 | 43.67 | 62.61 |
|  | 1 | 0.98 | 37.80 | 58.81 |
|  | 0.5 | 1.01 | 36.07 | 57.56 |
| PEG-4000+SDBS | 2 | 0.79 | 50.01 | 66.81 |
|  | 1.5 | 0.85 | 46.20 | 64.29 |
|  | 1 | 0.93 | 41.06 | 60.97 |
|  | 0.5 | 1.02 | 35.44 | 57.14 |
| Tween-80+SDBS | 2 | 0.77 | 51.27 | 67.65 |
|  | 1.5 | 0.87 | 44.94 | 63.45 |
|  | 1 | 0.98 | 37.90 | 58.88 |
|  | 0.5 | 1.04 | 34.18 | 56.30 |
| Tween-80+SLS | 2 | 0.78 | 50.63 | 67.22 |
|  | 1.5 | 0.87 | 44.94 | 63.45 |
|  | 1 | 0.96 | 39.05 | 59.63 |
|  | 0.5 | 1.01 | 36.08 | 57.56 |
